# A phase II randomised clinical trial comparing cisplatin, paclitaxel and ifosfamide with cisplatin, paclitaxel and epirubicin in newly diagnosed advanced epithelial ovarian cancer: long-term survival analysis

**DOI:** 10.1038/sj.bjc.6604231

**Published:** 2008-02-05

**Authors:** R Fruscio, N Colombo, A A Lissoni, A Garbi, R Fossati, N Ieda', V Torri, C Mangioni

**Affiliations:** 1 Clinica Ostetrica e Ginecologica, University of Milan-Bicocca, San Gerardo Hospital, Monza, Italy; 2 European Institute of Oncology, University of Milan-Bicocca, Milan, Italy; 3 Department of Oncology, Mario Negri Institute for Pharmacological Research, Milan, Italy

**Keywords:** ovarian cancer, first-line chemotherapy, epirubicin, ifosfamide, triplets, long-term survival

## Abstract

To test the feasibility and efficacy of epirubicin and ifosfamide added to first-line chemotherapy with cisplatin and paclitaxel in a phase II randomised clinical trial. Patients with histologically proven epithelial ovarian cancer were randomly assigned to receive first-line polychemotherapy with cisplatin/paclitaxel/epirubicin (CEP) or cisplatin/paclitaxel/ifosfamide (CIP) for six cycles every 21 days. Two hundred and eight patients were randomised between the two treatment arms and the median number of cycles per patient was six. Toxicity was predominantly haematological with both regimens; however, anaemia, leucopaenia, neutropaenic fever and use of granulocyte colony-stimulating factors and transfusion were significantly more frequent in the CIP treatment arm. Response rates were 85% (95% confidence interval (CI) 77–93%) in the CIP arm and 90% (95% CI 84–96%) in the CEP arm; complete response rates were 48 and 52%. After a median follow-up of 82 months, median overall survival (OS) was 51 and 65 months; 5-year survival rates were respectively 43 and 50%. In this clinical trial, both regimens showed good efficacy, but toxicity was heavier with the CIP regimen. Considering that more than 50% of patients were suboptimally debulked after the first surgery, OS seems to be longer than is commonly reported. This unexpected finding might be a consequence of the close surgical surveillance and aggressive chemotherapeutic approach.

Advanced epithelial ovarian cancer treatment is still a challenge for gynaecologic oncologists. Despite recent improvements in treatment, most ovarian cancer patients will relapse eventually, and only about one-third are alive after 5 years (Heintz *et al*, 2006).

Today, cytoreductive surgery followed by six cycles of carboplatin/paclitaxel is widely accepted as the standard therapy for advanced ovarian cancer (McGuire *et al*, 1996; du Bois *et al*, 1997; Neijt *et al*, 2000; Piccart *et al*, 2000). Attempts to improve long-term survival have involved the addition of a third non-cross-resistant drug with proven antitumour activity to first-line chemotherapy .

We report here a large phase II clinical trial that started in 1997 with the primary objective of exploring the effects in terms of toxicity, response to therapy and survival of the addition of epirubicin and ifosfamide to the cisplatin/paclitaxel regimen. Cisplatin was chosen because it was the standard platinum compound used in ovarian cancer when the trial started. A few years later, cisplatin was replaced by carboplatin, because it was better tolerated with an improved quality of life. Indeed two large, independent phase III trials conducted by AGO and GOG reported similar efficacy for the two drugs (du Bois *et al*, 2003; Ozols *et al*, 2003).

Until recently, epirubicin was considered an option mainly for the treatment of recurrent or platinum-resistant disease (Ray-Coquard *et al*, 2003; Buda *et al*, 2004), with similar antitumour activity but fewer side effects than doxorubicin (Maluf and Spriggs, 2002). Doxorubicin had a significant impact on the response rate and on the long-term overall survival (OS) when added to first-line chemotherapy with cisplatin (The Ovarian Cancer meta-analysis project, 1991; Fanning *et al*, 1992; A’Hern and Gore, 1995). Epirubicin too has been tested in several phase I/II trials in combination with paclitaxel and carboplatin at doses of 60 or 75 mg m^−2^, with promising results, the main toxicity of the combination being myelosuppression (Hill *et al*, 1997; Naumann *et al*, 1998; du Bois *et al*, 1999; Gregory *et al*, 2000; Papadimitriou *et al*, 2000; Fleming *et al*, 2001; Kristensen *et al*, 2003; Romanini *et al*, 2003). However, two recent randomised clinical trials found that the addition of epirubicin to standard treatment did not improve OS or progression-free survival (PFS) (Kristensen *et al*, 2005; du Bois *et al*, 2006).

The second drug we decided to study in this trial in addition to cisplatin and paclitaxel was ifosfamide. Ifosfamide has been studied in combination with other drugs such as etoposide, topotecan and vinorelbine for the treatment of recurrent epithelial ovarian cancer (Gonzalez-Martin *et al*, 2002; Chiara *et al*, 2004; Shaheen *et al*, 2004). Shortly after the start of this clinical trial, in a phase I and a phase II trial of cisplatin and paclitaxel combined with ifosfamide, significant activity against ovarian epithelial cancer was noted. The regimen was feasible with ifosfamide doses up to 5 g m^−2^ (Kosmas *et al*, 2001; Papadimitriou *et al*, 2001).

## PATIENTS AND METHODS

### Patients

Women 18 years of age or older with histologically proven epithelial ovarian carcinoma were recruited and randomly assigned to the cisplatin/paclitaxel/ifosfamide (CIP) or cisplatin/paclitaxel/epirubicin (CEP) treatment arm. Inclusion criteria were International Federation of Gynecologic Oncology stage II–IV, Eastern Cooperative Oncology Group performance status 0–2, no prior chemotherapy or radiotherapy, adequate haematologic, hepatic and renal function defined as absolute neutrophil count >1.5 × 10^9^ cells per litre, platelet count at least 100 × 10^9^ cells per litre, serum creatinine and total bilirubin not more than 1.25 times the upper normal limit.

Exclusion criteria included mixed mesodermal tumours, borderline tumours, concurrent malignancies within the previous 5 years (excluding basal cell carcinoma), pregnancy, lactation, peripheral neuropathy of grade 2 or higher, congestive heart failure and cardiac arrhythmias.

Patients were randomised within 4 weeks of surgery. The study had ethics committee approval from the two participating centres (San Gerardo Hospital, Monza, Italy and European Institute of Oncology, Milan, Italy), and all the patients gave written informed consent.

### Treatment plan

Six cycles of chemotherapy were planned with 3-week intervals between them, the first cycle starting within 2 weeks of randomisation.

In CEP, drug doses were epirubicin 80 mg m^−2^, paclitaxel 175 mg m^−2^ and cisplatin 75 mg m^−2^. Epirubicin was administered before paclitaxel (Venturini *et al*, 2000). In CIP, paclitaxel and cisplatin doses were the same as for CEP; ifosfamide was given at 5 g m^−2^ with the chemoprotectant MESNA.

Cycles were repeated if no progressive disease or prohibitive toxicity occurred. Treatment was delayed for up to 2 weeks if the neutrophil count was less than 1.5 × 10^9^ per litre and the platelet count was less than 100 × 10^9^ per litre on day 1 of each cycle.

Dose reductions were allowed in any of the following circumstances: 1-week cycle delay for two cycles in succession or 2-week cycle delay because of prolonged bone marrow depression; febrile neutropaenia or thrombocytopaenic bleeding; any drug-related side effect requiring hospitalisation with i.v. antibiotics or platelet transfusion. Dose-reduction levels were epirubicin 60 mg m^−2^ (level 1) and 45 mg m^−2^ (level 2), ifosfamide 2.5 g m^−2^ (level 1) and 1.5 g m^−2^ (level 2), cisplatin 50 mg m^−2^ (level 2) and paclitaxel 135 mg m^−2^ (level 2).

Patients with a partial response after the six cycles of chemotherapy could receive additional treatment cycles or a second chemotherapeutic regimen, at the investigator's discretion.

### Clinical assessment

CA125 levels were measured before each cycle. Response was assessed with a CT scan after the third and the last cycles. The CA125 response was classified according to the method of Rustin *et al* (1996). Clinical response was assessed using WHO criteria (Miller *et al*, 1981). Adverse events and toxicity were graded using the National Cancer Institute Common Toxicity Criteria version 2.0 (National Cancer Institute). Second-look surgery was permitted.

Follow-up for each patient consisted of a physical examination every 3 months for the first 3 years after chemotherapy, every 6 months in the next 2 years and one visit every year thereafter. CA125 was measured before each visit. Computed tomography was repeated yearly for 5 years, if there was any suspicion of relapse or progressive disease.

### Statistical analysis

This was a phase II, multicentre, randomised clinical trial. Sample size was based on the assumption of a pathologic complete response (pCR) rate of 20% with standard treatment, and was planned to exclude a pCR <15% with *a*=0.05, and to recognise a pCR=30% with a power=0.85. According to these criteria, 51 evaluable patients per arm (60 randomised) should have been entered. Data of this phase II study have been published (Colombo *et al*, 1999), leading to the following conclusions: (1) both regimens were feasible and (2) pCR rates were higher than those expected with other standard therapies. Therefore, based on these positive evidences, it was decided to continue the study to confirm positive response rates in a larger sample and to obtain long-term survival data and further confirmatory evidence to move a regimen into a phase III trial. Sample size was based on the assumption that the historic median survival is 36 months in patients receiving the reference platinum and paclitaxel doublet regimen (CP). Given this assumption and that a 33% improvement in 3-year survival (hazard ratio=0.66, which translates into increases from 50 to 63% in 3-year survival) was considered as sufficient evidence to move a regimen into a phase III trial, each treatment arm was planned to have about 100 patients (type-I error limited to 0.05 (one-tailed test); power 0.80). For this further analysis, primary end point was OS, defined as the time from randomisation to death from any cause. Secondary end points were PFS, defined as the time from randomisation to the earliest occurrence of progression or death from any cause and overall response rate. Other secondary outcome measures aimed to assess safety included frequency and severity of adverse events. Patients meeting all inclusion criteria were consecutively randomised by a central data centre.

This study was noncomparative and was not powered to demonstrate differences between treatment arms. Although no formal statistical comparison of the two arms was planned, survival curves were estimated by the Kaplan–Meier method and compared using the log-rank test. Additional time-to-event analyses were done using the Cox proportional hazards model, adjusting for multiple baseline characteristics. Fisher's exact test was done on the response rates and toxicity levels. These tests were for exploratory purposes only, and all *P*-values are two-sided; statistical significance was set at 0.05 and analysis was done using SAS software version 9.0 (SAS Institute Inc., Cary, NC, USA).

## RESULTS

### Population

Two hundred and eight patients were randomised between the two treatment arms (106 to CIP and 102 to CEP) in the two recruiting centres (San Gerardo Hospital, Monza and European Institute of Oncology, Milan). One patient allocated to the CEP arm was ineligible because of a diagnosis of pancreatic metastatic carcinoma. One patient allocated to the CIP arm died before receiving any chemotherapy. Three patients allocated to CIP and three to CEP received only platinum and paclitaxel. One patient allocated to the CIP arm refused the chemotherapy. Therefore 199 patients were eligible for safety and efficacy assessment (101 in the CIP arm and 98 in the CEP arm).

The two treatment arms were well balanced with respect to baseline characteristics (Table 1).

### Toxicity

A total of 1149 cycles of chemotherapy were administered (575 CIP, 574 CEP), and toxicity data were available for 85% of the cycles; maximum toxicity grades were available for all the patients.

Treatment changes because of toxicity for each drug are shown in Table 2. In the CIP arm, 38% of patients received the planned dose of ifosfamide, 46% received a one-level reduction and 16% a two-level reduction. In the CEP arm, 51% of patients received the full dose of epirubicin, 46% received a one-level dose reduction and 3% a two-level dose reduction. One patient in the CEP arm had to discontinue epirubicin on account of a generalised maculo-papular rash, and in the CIP arm, ifosfamide was suspended after the third cycle for a worsening of chronic hepatitis.

Haematological toxicity is summarised in Table 3. Nonhaematological toxicity is shown in Table 4.

Allergic reactions were all to paclitaxel and occurred in 23 patients in all; 21 consisted only of short-lasting flushing without fever (grade 1), while one patient in the CIP arm experienced a grade 3 (oedema) and one a grade 4 (anaphylaxis) allergic reaction. Both these patients were able to receive the remaining cycles of chemotherapy after the adverse event, with a change in premedication. There were two cases of cardiovascular toxicity (one had tachycardia and one a cardiac ischaemia that did not require intervention) in the CIP arm only. The two patients in the CEP arm who had grade 2 vascular toxicity had superficial phlebitis, while the only patient with grade 4 toxicity had pulmonary thromboembolism.

### Response rates

Clinical and pathological responses to treatment are summarised in Table 5. Of the 191 patients with stage III–IV disease who received at least two cycles of chemotherapy, 172 were assessable for response. Response was not evaluable in 19 patients (11 in the CIP arm and 8 in the CEP arm) because they had no evidence of disease after the first surgery and no positive CA125 levels at the start of chemotherapy. Of the 123 patients with suboptimal debulking (residual tumour (RT) >1 cm) after first surgery, 95 underwent second-look laparoscopy after the planned course of chemotherapy. Thirty-three of the 68 patients with microscopic or no residual disease after the first surgery underwent the same procedure; thus pathological response could be evaluated on 128/191 patients (67%). Responses of the remaining 44 patients (28 with measurable disease and 16 with positive CA125 only) were evaluated on the basis of clinical, radiological and biochemical data.

Overall, 38 patients had a clinical complete/partial response, while 113 had a pathological complete/partial response.

Response rates were 85% (95% CI 77–93%) in the CIP arm and 90% (95% CI 84–96%) in the CEP arm; complete response rates were 48 and 52%.

### Survival analysis

**Figure 1:**
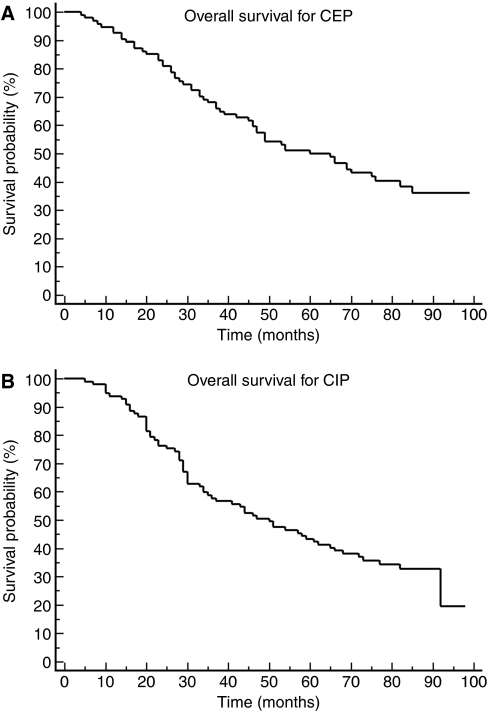
(**A**) Overall survival for the CEP regimen. (**B**) Overall survival for the CIP regimen.

**Figure 2:**
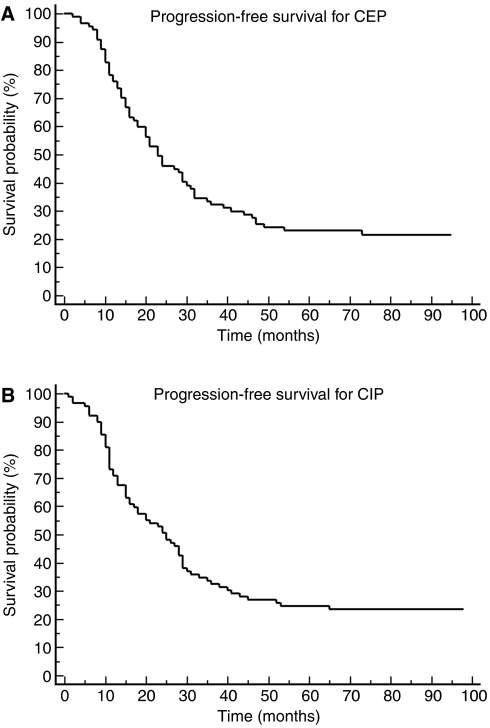
(**A**) Progression-free survival for the CEP regimen. (**B**) Progression-free survival for the CIP regimen

Overall survival was analysed excluding the eight patients with stage II ovarian cancer. No patient was lost to follow-up. Therefore, survival data were available for a total of 191 patients (97 in the CIP arm and 94 in the CEP arm). Median follow-up was 82 months. Median OS was 51 months in the CIP arm and 65 months in the CEP arm (Figure 1A and B). Median OS was 54 months for all patients, and 57 months including the eight patients with stage II disease. Median OS was only 43 months for patients who had been suboptimally debulked. Patients with RT >5 cm after first surgery had OS 31 months (33 in the CEP arm and 30 months in the CIP arm). Median PFS was 25 months in the CIP arm and 23 months in the CEP arm (Figure 2A and B). Overall survival at the third and the fifth year of the patients on CIP was 59% (95% confidence interval (CI) 48–69%) and 43% (95% CI 33–53%), and for patients on CEP was 68% (95% CI 58–77%) and 50% (95% CI 40–60%), respectively. Although both regimens were active, only CEP regimen would formally merit further study as the third year OS for this regimen was significantly better than historical rate (i.e. the lower CI of third year OS was higher than 50%).

## DISCUSSION

One of the secondary objectives of this study was to compare the toxicity of the two experimental regimens and assess their feasibility, because one of the main risks of adding a third drug to standard chemotherapy is a predictable increase in adverse events.

Overall, both the combinations used in this phase II clinical trial showed good feasibility and acceptable tolerability and toxicity, especially CEP, using the two drugs under investigation at relatively high doses. As expected from published toxicity data, the CIP arm was more toxic than the CEP arm. More than 60% of patients who received CIP did not tolerate the starting dose of 5 g m^−2^ ifosfamide and required at least one dose-level reduction. However, even in the CEP arm, almost half the patients could not complete the six courses of chemotherapy with the planned doses of the three drugs.

In our opinion, the main limitation to CIP polychemotherapy at the doses presented in this trial is that its toxicity seems less manageable than with CEP, as indicated by the higher frequency of leucopaenia (*P*=0.0001), anaemia (*P*=0.002), need for granulocyte colony-stimulating factor (G-CSF) (*P*=0.0002) and transfusion (*P*=0.03) and, above all, the higher incidence of febrile neutropaenia, considered as a potentially life-threatening event. The subsequent need for hospitalisation, besides worsening patients’ quality of life, raises the costs of therapy.

Toxicity in the CIP arm was greater than reported elsewhere; however, the phase I dose-escalating study (Papadimitriou *et al*, 2001) used prophylactic G-CSF and had febrile neutropaenia in one patient out of eight treated at doses comparable with ours, and the phase II trial (Kosmas *et al*, 2001) reported less frequent high-grade haematological toxicity but used only 1.5 g m^−2^ ifosfamide, which is 70% lower than the dose we used. Overall, the results of this study indicate low feasibility of the CIP regimen with a starting dose of 5 g m^−2^ ifosfamide, at least without prophylactic G-CSF.

On the other hand, most patients recovered from the toxicity induced by CEP with a dose reduction and a week's delay of therapy. The heavier toxicity of CIP would only be justified by greater efficacy than CEP or the standard chemotherapy. However, the two regimens showed similar results in terms of response rates.

As expected, response rates obtained in this trial are similar to those reported elsewhere.

Although no formal comparison of the two regimens was done in terms of OS, only the CEP regimen showed a significant improvement over historical therapy in the 3-year survival rate, which was the formal target outcome for our experimental design. However, it was the long-term survival in both study arms, which was noteworthy on account of the absence of patients lost to follow-up and the unusually long median follow-up (82 months), that merits comment. Median OS for all patients with stage III–IV epithelial ovarian cancer treated in the two arms was 54 months, and 62 months in the CEP arm. This is particularly interesting because 65% of patients in our study had an RT after first surgery >1 cm. Median OS for patients with an RT <1 cm has not yet been reached in either group, while it was 44 months for patients with an RT >1 cm.

Even if this study had no control arm with the standard carboplatin–paclitaxel chemotherapy, making it impossible to compare the OS obtained with our experimental regimens, we did try to compare the results with some of the major randomised clinical trials published in the last 20 years (Table 6).

Since the introduction of platinum compounds in first-line chemotherapy, PFS has not improved much over the years, but OS has risen from about 20 months in the 1980s (Conte *et al*, 1986; Alberts *et al*, 1992; Rothenberg *et al*, 1992; Swenerton *et al*, 1992; Alberts *et al*, 1996) to 30 months in the 1990s (McGuire *et al*, 1996; ICON Collaborators, 1998; Muggia *et al*, 2000) and 40 months in the last few years (Markman *et al*, 2001; ICON Collaborators, 2002; Du Bois *et al*, 2003; Armstrong *et al*, 2006). In our series, PFS was slightly better than is commonly reported and considering the composition of our population, OS of 54 months is remarkable. Comparable results are reported only in trials that enrolled only patients with minimal or no residual disease after the first surgery (Alberts *et al*, 1996; Markman *et al*, 2001; ICON Collaborators, 2002; Armstrong *et al*, 2006), and in other studies that considered the subgroup of patients with this positive prognostic factor (du Bois *et al*, 2006; Pfisterer *et al*, 2006).

Interestingly, a recently published study on the role of aggressive cytoreductive surgery in the treatment of advanced ovarian cancer reports a 5-year survival rate of 46% for patients who underwent radical surgery, similar to the present result (Eisenkop *et al*, 2003; Aletti *et al*, 2006).

However, some recently published randomised clinical trial (Bookman *et al*, 2006; du Bois *et al*, 2006; Pfisterer *et al*, 2006) failed to demonstrate an advantage for the triplets in the treatment of ovarian cancer patients compared to the standard doublet, showing an increased toxicity associated with the experimental treatment arms. Therefore, considering that our populations had no favourable clinical or biological prognostic factors, one of the reasons for better oncological outcome in our study might be the diagnostic and therapeutic procedures that followed first-line chemotherapy. Second-look laparoscopy was done in 67% of patients, and patients with persistent disease received a second chemotherapy course. After second-line chemotherapy laparoscopy was repeated, and the treatment was continued until pathological evidence of complete response. Of the 120 patients alive after 3 years, only 23% had not received any further chemotherapy after the first-line, whereas 42% received three or more different regimens; this proportion reaches 53% among patients with suboptimal debulking.

This last observation suggests that the long PFS and OS of our patients might have been attributed to the chronicity of the postsurgical therapies, despite the fact that consolidation therapy of ovarian cancer has not been proven to increase survival. In addition, the impact of chronic chemotherapies on the quality of life of patients with ovarian cancer needs to be performed in future studies.

Another factor that might have influenced our results is the cumulative dose of cisplatin. Following the trial protocol, we tried to give all patients the full platinum dose, reducing the two experimental drugs epirubicin and ifosfamide by one-dose levels in case of toxicity without recovery before reducing cisplatin and paclitaxel.

In conclusion, both CIP and CEP showed good efficacy during long-term follow-up, but toxicity was greater than indicated by historical data with the standard carboplatin/paclitaxel chemotherapy. This observation adds to the evidence that adding a third drug to the standard chemotherapy does increase the toxicity but gives no clear advantage in efficacy. By integrating information such as OS and toxicity, the combination of cisplatin, paclitaxel and epirubicin we used should be chosen over CIP regime. Cisplatin/paclitaxel/epirubicin arm gave a very high 5-year survival rate, but in the absence of a comparison with current standard treatment, it cannot be recommended as first-line chemotherapy for advanced ovarian cancer.

## Figures and Tables

**Table 1:** Patients' main characteristics

	**CIP (*n*=101)**	**CEP (*n*=98)**
	**%**	**%**
*Age (years)*		
Median	52	52
Range	27	24
	73	71
		
*BSA (m^2^)*		
Median	1.6	1.6
Range	1.3	1.3
	2.4	2.0
*PS*		
0	89	92
1	9	8
2	2	0
		
*FIGO stage*		
IIA	0	2
IIB	3	1
IIC	1	1
IIIA	1	3
IIIB	6	4
IIIC	76	74
IV	13	15
		
*Histology*		
Serous	64	70
Mucinous	4	3
Endometrioid	19	10
Clear cells	5	7
Poorly differentiated	8	10
		
*Grade*		
1	4	6
2	20	14
3	76	80
		
*Residual tumour*		
NED	20	15
< 1 cm	14	24
1–2 cm	14	11
2–5 cm	23	18
5–10 cm	11	19
>10 cm	18	13
		
*Type of surgery* a		
1	30	17
2	44	52
3	19	23
4	7	8

Abbreviations: BSA=body surface area; CEP=cisplatin/paclitaxel/epirubicin; CIP=cisplatin/paclitaxel/ifosfamide; FIGO=Federation of Gynecologic Oncology; NED=no evidence of disease; PS=performance status.

a 1=laparotomic histerectomy, bilateral annesiectomy, omentectomy, appendicectomy, pelvic linphoadenectomy; 2=laparotomic histerectomy, bilateral annesiectomy, omentectomy, appendicectomy; 3=laparotomic histerectomy, bilateral annesiectomy; 4=biopsies only.

**Table 2:** Treatment modifications and toxicity management in both arms

	**CIP (*n*=101)**	**CEP (*n*=98)**	
**Toxicity**	**%**	**%**	***P***
Number of cycles administered	575	574	
Number of patients delayed	49	37	0.08
Weeks of treatment delay, total	116	117	
Cisplatin reduction	21	22	0.81
Paclitaxel reduction	27	17	0.09
Epirubicin/ifosfamide reduction	62	49	0.07

Abbreviations: CEP=cisplatin/paclitaxel/epirubicin; CIP=cisplatin/paclitaxel/ifosfamide.

**Table 3:** Grade 3–4 haematological toxicity and management in both arms

	**CIP (*n*=101)**	**CEP (*n*=98)**	
**Toxicity**	**%**	**%**	***P***
Anaemia	48	27	0.002
Leukopaenia	95	76	0.0001
Neutropaenia	97	95	0.51
Thrombocytopaenia	37	25	0.08
Febrile neutropaenia, grade 3	14	3	0.007
G-CSF	48	22	0.0002
Transfusion	37	22	0.03
Hospitalisation	34	19	0.02

Abbreviations: CEP=cisplatin/paclitaxel/epirubicin; CIP=cisplatin/paclitaxel/ifosfamide; G-CSF=granulocyte colony-stimulating factors.

**Table 4:** Nonhaematological toxicity in both treatment arms

	**CIP (*n*=101)**		**CEP (*n*=98)**	
	**Grade 1–2**	**Grade 3–4**	**Grade 1–2**	**Grade 3–4**
**Toxicity**	**%**	**%**	**%**	**%**
Allergy	13	2	8	0
Cardiac	1	1	0	0
Vascular	0	1	2	0
Artromyalgia	37	5	39	5
Nausea	78	21	76	24
Vomiting	73	24	80	17
Mucositis	43	3	53	2
Fever^a^	38	2	19	0
Infection	16	2	15	2
Neurotoxicity	59	3	69	0
Alopaecia	0	98	0	98

Abbreviations: CEP=cisplatin/paclitaxel/epirubicin; CIP=cisplatin/paclitaxel/ifosfamide.

a Statistically significant difference between the two treatment regimens (*P*<0.01).

**Table 5:** Response to treatment for patients with stage III–IV disease

	**CIP (*n*=97)**	**CEP (*n*=94)**
**Response**	**No. (%)**	**No. (%)**
Complete response	41 (48)	45 (52)
Clinical	12 (14)	14 (16)
Pathological	29 (34)	31 (36)
Partial response	32 (37)	33 (38)
Clinical	4 (5)	8 (9)
Pathological	28 (33)	25 (29)
Stable disease	10 (12)	7 (8)
Clinical	3 (3)	1 (1)
Pathological	7 (8)	6 (7)
Progressive disease	3 (3)	1 (1)
Clinical	2 (2)	0
Pathological	1 (1)	1 (1)
Inevaluable^a^	11	8

Abbreviations: CEP=cisplatin/paclitaxel/epirubicin; CIP=cisplatin/paclitaxel/ifosfamide.

a Response was not evaluable in patients without evidence of disease after the first surgery and negative CA125.

**Table 6:** Historical OS and PFS for patients with advanced ovarian cancer

**First author**	**Journal**	**Patients**	**Drugs**	**PFS^a^**	**OS^a^**
Conte PF	*J Clin Oncol* (1986) **4**: 965–969	125	CDDP+CTX	12.6	22.7
			CDDP+CTX+Doxo	13.3	26.7
Alberts DS	*J Clin Oncol* (1992) **10**: 706–710	342	CDDP+CTX	N/A	17.4
			CBDCA+CTX	N/A	20
Swenerton K	*J Clin Oncol* (1992) **10**: 718–721	447	CDDP+CTX	13.1	23.3
			CBDCA+CTX	13.5	25.7
Rothenberg ML	*J Clin Oncol* (1992) **10**: 727–734	50	CDDP+CTX+RT	N/A	23.4
McGuire WP	N Engl J Med (1996) **334**: 1–6	235	CDDP+CTX	13	24
			CDDP+PTX	18	36
Alberts DS	*N Engl J Med* (1996 Dec 26) **335**(26): 1950–1955.	546	CDDP+CTX	23.8	49
			CDDP+CTX	18.3	41
ICON	*Lancet* (1998) **352**: 1571–1576.	1526	CBDCA	15.5	33
			CPPD+CTX+Doxo	17	33
Muggia FM	*J Clin Oncol* (2000 Jan) **18**(1): 106–115.	648	CDDP	16.4	30.2
			PTX	10.8	25.9
			CDDP+PTX	14.1	26.3
Markman M	*J Clin Oncol* (2001 Feb 15) **19**(4): 1001–1007	462	CDDP+PTX	22	52
			CBDCA+PTX+CDDP	28	63
ICON	*Lancet* (2002 Aug 17) **360**(9332): 505–515	2074	CBDCA+PTX	17.3	36.1
			CPPD+CTX+Doxo	16.1	35.4
Du Bois A	*J Natl Cancer Inst* (2003 Sep 3) **95**(17): 1320–1329.	798	CBDCA+PTX	17.2	43.3
			CDDP+PTX	19.1	44.1
Ozols RF	*J Clin Oncol* (2003 Sep 1) **21**(17): 3194–3200	792	CBDCA+PTX	20.7	57.4
			CDDP+PTX	19.4	48.7
Vasey PA	*J Natl Cancer Inst* (2004 Nov 17) **96**(22): 1682–1691	1077	CBDCA+PTX	14.8	N/A
			CBDCA+DTX	15	N/A
Pfisterer J	*J Natl Cancer Inst* (2006 Aug 2) **98**(15): 1036–1045.	1308	CBDCA+PTX	18.5	43.1
			CBDCA+PTX+TPT	18.2	44.5
du Bois A	*J Clin Oncol* (2006 Mar 1) **24**(7): 1127–1135.	1282	CBDCA+PTX	17.9	41
			CBDCA+PTX+Epi	18.4	45.8
Bookman MA	*J Clin Oncol* (2006) ASCO Annual Meeting Proceedings Part I. Vol 24, No. 18S (June 20 Supplement), 2006: 5002	4312	CBDCA+PTX	16.1	40
			CBDCA+PTX+Gem	16.4	40.4
			CBDCA+PTX+Doxo	16.4	42.8
			CBDCA+PTX+TPT	15.3	39.1
			CBDCA+PTX+Gem	15.4	40.2
Armstrong DK	*N Engl J Med* (2006 Jan 5) **354**(1): 34–43	415	CDDP+PTX	23.8	65.6
			CDDP+PTX	18.3	49.7

Abbreviations: CBDCA=carboplatin; CDDP=cisplatin; CTX=cyclophosphamide; Doxo=doxorubicin; DTX=docetaxel; Epi=epirubicin; Gem=gemcitabine; OS=overall survival; PFS=progression-free survival; PTX=paclitaxel; RT=radiotherapy; TPT=topotecan.

a PFS and OS are expressed in months.
